# Rare and life-threatening iliac vein stent infection following radiotherapy: a case report emphasizing clinical urgency and preoperative stent evaluation

**DOI:** 10.3389/fcvm.2025.1532300

**Published:** 2025-04-28

**Authors:** Jianyu Liao, YuKui Ma, Zhoupeng Wu, Xinyan Wang, Jichun Zhao

**Affiliations:** Division of Vascular Surgery, Department of General Surgery, West China Hospital, Sichuan University, Chengdu, China

**Keywords:** iliac vein stent, venous stent infection, radiotherapy complications, cervical cancer, colonic fistula

## Abstract

This case report discussed a 43-year-old female who underwent multiple radiotherapy sessions after cervical cancer surgery and experienced serious complications due to simultaneous iliac vein stent placement immediately after thrombus aspiration without adequate evaluation of the indication for stent placement. Two months after radiotherapy, the patient developed right lower limb edema and pain, which led to the discovery of an iliac vein thrombosis. Subsequent stent placement without thorough evaluation resulted in severe complications, including infection and sepsis. Despite initial symptom relief, the patient was readmitted with high fever and severe pain, and imaging revealed gas around the stent, indicating infection. An exploratory laparotomy uncovered a large abscess and a colonic fistula. The stents were removed, and the patient underwent aggressive anti-infection treatment involving meropenem and vancomycin, along with surgical repair of the fistula. This case highlights the importance of accurate diagnosis and careful consideration of stent placement in preventing severe outcomes, including the rare but serious risk of venous stent infections requiring surgical intervention.

## Introduction

Post-radiotherapy complications in cervical cancer patients can be complex and challenging to diagnose. Radiotherapy, while effective in treating malignancies, can cause significant damage to surrounding tissues, leading to complications such as fibrosis, abscess formation, and vascular compression, although these complications are very rare (1). Patients often present with non-specific symptoms such as nausea, vomiting, and abdominal pain, which can obscure more serious underlying conditions (2). Accurate diagnosis and comprehensive preoperative assessments, including advanced imaging techniques like CT scans, are crucial to identify these complications early and plan appropriate interventions.

Additionally, patients with gynecologic malignancies often enter a hypercoagulable state following surgery, rendering them particularly susceptible to deep vein thrombosis (DVT) (3). The use of chemotherapy agents can further exacerbate this risk by contributing to endothelial damage and dysregulation of coagulation pathways (3). Consequently, when such patients develop DVT alongside the rare complications discussed above (e.g., fibrosis or abscess formation after radiotherapy)—the latter of which can compress the iliac vein and further exacerbate DVT—the clinical scenario becomes markedly more complex (4). Determining whether to recanalize the deep venous system and proceed with iliac vein stent placement can pose significant challenges, and inadequate evaluation may lead to severe consequences. This complexity underscores the importance of meticulous preoperative planning and individualized treatment strategies, as illustrated by the case presented in this report.

## Case presentation

A 43-year-old female patient previously treated with a total hysterectomy and bilateral adnexectomy for cervical cancer underwent three chemotherapy cycles and 28 radiation therapy sessions. Throughout the radiation therapy, she frequently reported nausea, vomiting, and abdominal pain, in addition to unexplained fevers peaking at 39°C. Two months following the completion of radiation, she developed right lower limb edema with pain. Ultrasound imaging performed at a local hospital suggested right external iliac vein thrombosis. An intervention under local anesthesia included catheter-directed thrombolysis which revealed “compression of the right iliac vein,” leading to a series of procedures: inferior vena cava angiography, filter placement, deep venous aspiration thrombectomy, right iliofemoral vein balloon dilation, and iliofemoral venous stent placement. Although the procedures initially alleviated her symptoms, the patient discontinued rivaroxaban one month later due to gastrointestinal bleeding. She was subsequently admitted to our hospital with recurrent right lower limb swelling and severe pain, alongside a fever of 42°C.

**Figure 1:**
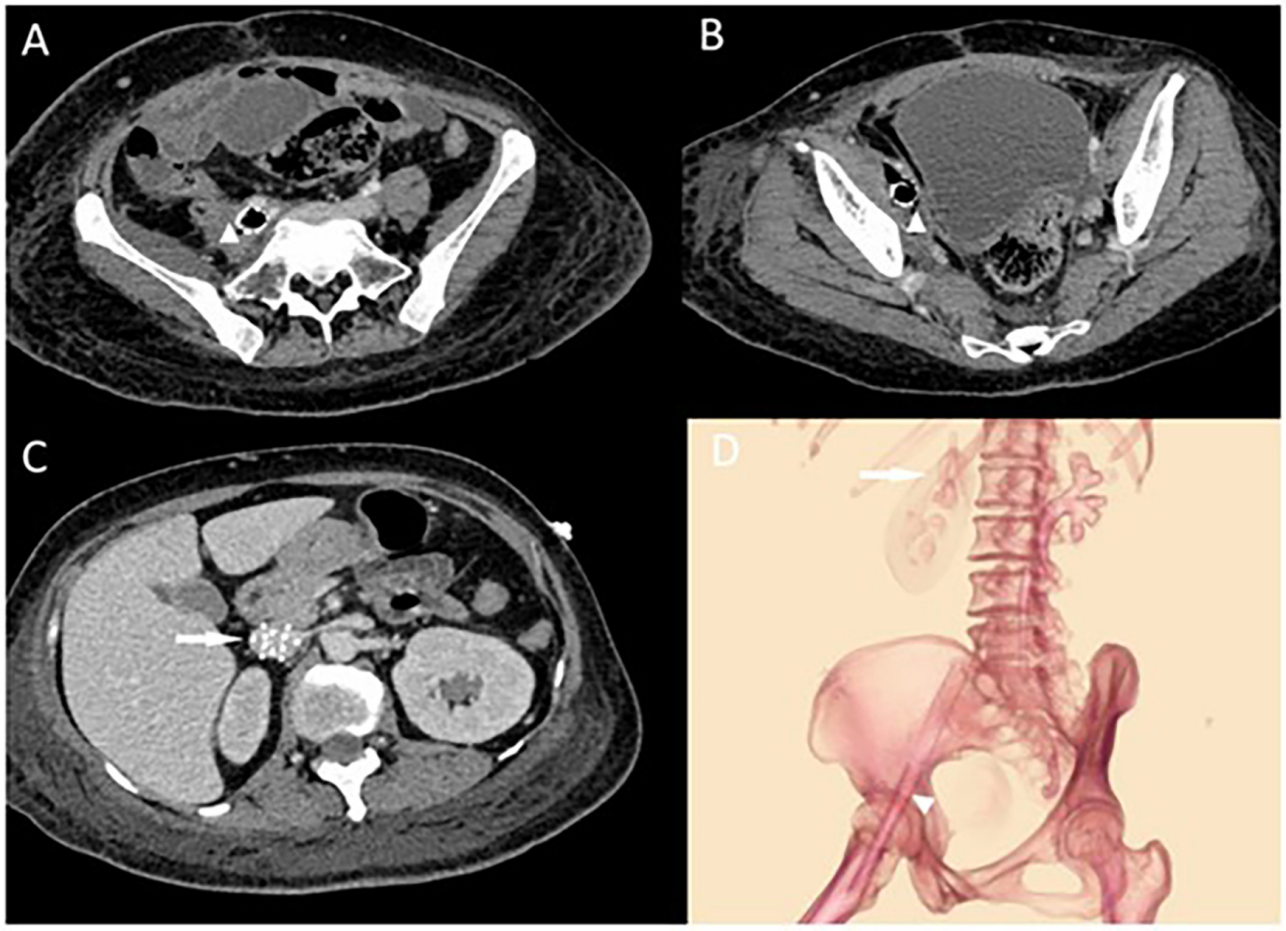
**(A,B)** Abdominal CT showing right external iliac vein and femoral vein stent with pneumatization and surrounding soft tissue swelling with pneumatization. The triangular markers indicate the abscess and the changes in the surrounding soft tissues around the stents. In **(C)**, the arrow points to the locally placed inferior vena cava (IVC) filter situated within the IVC. **(D)** Illustrates the positions of the IVC filter and the two stents, showing their course crossing over the inguinal ligament.

Laboratory tests revealed a normal white blood cell count (WBC) of 6.7 × 10⁹/L despite markedly elevated infection markers, including C-reactive protein (CRP) at 74.2 mg/L, interleukin-6 (IL-6) at 79.3 pg/ml, and procalcitonin (PCT) at 73.8 ng/ml, suggestive of a severe systemic inflammatory response. Meanwhile, pancytopenia was observed, with hemoglobin (Hb) at 51 g/L and platelet count (PLT) at 44 × 10⁹/L. Physical examination identified moderate pitting edema in the right lower limb and the foot below the left ankle. Blood cultures indicated dual infection with Escherichia coli and Enterococcus faecalis. Venous ultrasound suggested thrombosis in the right femoral and superficial femoral veins. An abdominal CT showed gas accumulation around the stent in the right external iliac and femoral veins with surrounding soft tissue swelling (Figure 1). We suspected an iliac vein stent infection leading to sepsis and planned an exploratory laparotomy to identify and manage the source of infection. Importantly, we decided against removing the filter during this hospital stay, planning its removal after assessing the risk of pulmonary embolism.

**Figure 2:**
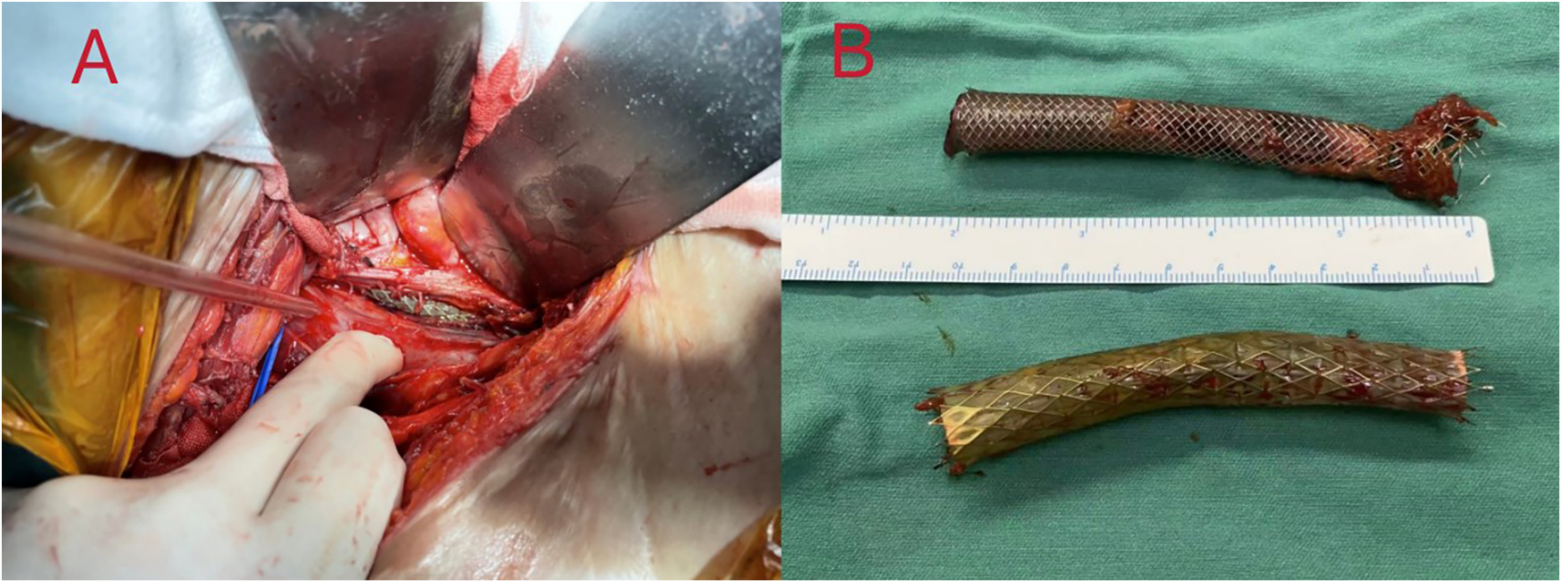
**(A)** after aspiration of the pus by dissection, an incision of the iliac vein was seen to expose the venous stent. **(B)** Complete removal of two different types of venous stents, which were seen to be covered with a large amount of pus moss. The covered stent is a GORE® VIABAHN® 10 mm × 60 mm, and the bare stent is a Wallstent® 12 mm × 60 mm.

Intraoperatively, a large abscess was discovered in the right iliac fossa, containing pus-soaked iliofemoral vein stents with thrombosis inside the stent and the adjacent vein (Figure 2). A 2 cm fistula was identified in the right wall of the rectosigmoid junction, leaking feces. We removed the iliofemoral vein stents, ligated the common iliac and femoral veins, repaired the colonic fistula, and created a colostomy. Postoperative management in the ICU involved aggressive anti-infection treatment initially with meropenem (1 g every 8 h) and vancomycin (1 g every 12 h), later stepped down to piperacillin-tazobactam (4.5 g every 8 h) after improvement of infection markers. Anticoagulation therapy began with fondaparinux sodium 2.5 mg daily starting on postoperative day 7 and gradually transitioned to rivaroxaban 20 mg daily at one month postoperatively, with dynamic monitoring and dose adjustment based on coagulation risk assessment. At a three-month follow-up, she was afebrile with no new DVT detected (Table 1).

**Table 1:** Timeline of the patient's clinical course and key interventions.

Timepoint	Event
7 months before admission	Underwent hysterectomy and bilateral salpingo-oophorectomy, followed by 3 cycles of chemotherapy and 28 sessions of radiotherapy.
5 months before admission	Developed persistent high fever (>39 °C) without an apparent cause.
3 months before admission	Presented with lower extremity swelling and underwent venography, resulting in the placement of a vena cava filter and iliac vein stent.
Day 2 after admission	Emergency surgery was performed, and the patient was transferred to the intensive care unit (ICU).
Postoperative Day 1	Successfully extubated and resumed spontaneous breathing; body temperature normalized.
Postoperative Day 6	Transferred back to the general ward.
Postoperative Day 20	Discharged and referred to a local hospital for continued care.
Three-month follow-up	Afebrile with no new deep vein thrombosis detected.

## Discussion

This case highlights the complexities of diagnosing and managing post-radiotherapy complications in cervical cancer patients, particularly when common post-treatment symptoms such as abdominal pain might obscure underlying developments like pelvic abscesses. These complications necessitate a high degree of vigilance. The abdominal pain in this case may have initially been linked to a pelvic abscess, which may have already formed during radiotherapy, subsequently leading to vein compression (5). This oversight emphasizes the need for careful consideration of radiotherapy-related complications. In this instance, it precipitated significant damage to colonic tissue, leading to a complex pelvic infection and the formation of a colonic fistula. And the hypercoagulable state in postoperative cervical cancer patients inherently presents a high risk for deep vein thrombosis (DVT), further obscuring the investigation into the underlying causes of DVT (6). These diagnostic oversights resulted in an incorrect assessment and the inappropriate placement of a venous stent, which subsequently caused severe complications, underscoring the critical need for accurate diagnosis and intervention planning.

Moreover, the involvement of the iliac vein and the subsequent infection of its stent draws attention to the differential diagnosis of iliac vein compression, including May-Thurner Syndrome (also known as Iliac Vein Compression Syndrome, IVCS). Traditionally associated with left-sided iliac vein compression, IVCS is increasingly recognized as affecting the right side and contributing to clinical symptoms (7). Additionally, it is important to note that while venography can diagnose iliac vein compression and is considered the gold standard for diagnosing pelvic congestion syndrome, its diagnostic accuracy and specificity are still lower compared to vascular ultrasound examinations. It often cannot clearly identify the extrinsic compression on the venous lumen (8). In this case, although guidelines mention that venous stents can extend across the inguinal ligament when the lesion is long and involves the deep femoral vein, there was no clear evidence indicating that the lesion length necessitated such an extended stent placement (9). However, the local operation records confirmed that the initial placement of the stent was appropriate; however, we believe that subsequent infection led to bacterial adhesion and gas formation, causing stent retraction and displacement to an “unreasonable” position across the inguinal ligament. For patients with an existing stent infection, the literature indicates that surgical removal of the stent is typically necessary. Although venous stent infections are rare, when they do occur, they are serious and often resistant to antibiotic treatment (10, 11). In this case, as in the few other reported cases of iliac vein stent infection primarily caused by Staphylococcus epidermidis from the skin, aggressive antibiotic treatment was not effective (12). Thus, the stent was surgically removed. Unlike arterial stents, which are more prone to hematogenous infections, venous stent infections are exceedingly rare. However, when they do occur, they similarly require removal to effectively manage the infection.

In this patient's case, the hysterectomy and bilateral adnexectomy indirectly resulted in a higher dose of postoperative radiotherapy to the rectum and colon, rendering that region more vulnerable to bacterial overgrowth. After more than 20 radiotherapy sessions, the bowel segment sustained sufficient damage to develop radiation enteritis and, eventually, a chronic fistula, which likely explains the patient's recurrent high fevers without a clear trigger prior to DVT onset. Moreover, the associated pelvic abscess caused by the fistula may have further compressed the iliac vein, thereby exacerbating the DVT. Although subsequent stent placement in the iliac vein briefly alleviated her symptoms, its proximity to the fistula introduced a foreign body into an already compromised region, facilitating bacterial colonization and precipitating severe sepsis. The intestinal origin of this infection was confirmed by blood cultures revealing Escherichia coli and Enterococcus faecalis, consistent with a gastrointestinal source. Consequently, opting for one-stage iliac vein stent placement in this setting was not appropriate.

## Conclusion

When dealing with patients with high fever and signs of infection after venous stent surgery, we should consider the possibility of a rare venous stent infection. Analyzing the cause of the infection is necessary. Identifying the primary site of infection and route of transmission is crucial for subsequent infection eradication, and prompt removal of the stent to eliminate the infection source is an effective and feasible approach. For cancer patients, stent placement should be approached with extreme caution. Comprehensive pre-operative assessments, such as CT scans to accurately define pelvic and abdominal anatomy, are essential (2). Relying solely on venography to suggest iliac vein compression as a basis for stent placement can lead to tragic outcomes, causing significant patient harm. Ensuring thorough and accurate diagnostic workup is imperative to prevent such severe complications and ensure better patient outcomes.

## Data Availability

The original contributions presented in the study are included in the article/Supplementary Material, further inquiries can be directed to the corresponding author.

## References

[1] Tirumani SH Baez JC Jagannathan JP Shinagare AB Ramaiya NH. Tumor-bowel fistula: what radiologists should know. Abdom Imaging. (2013) 38(5):1014–23. 10.1007/s00261-013-9987-6 23455947

[2] Loge L Florescu C Alves A Menahem B. Radiation enteritis: diagnostic and therapeutic issues. J Visc Surg. (2020) 157(6):475–85. 10.1016/j.jviscsurg.2020.08.012 32883650

[3] Caine GJ Stonelake PS Lip GY Kehoe ST. The hypercoagulable state of malignancy: pathogenesis and current debate. Neoplasia. (2002) 4(6):465–73. 10.1038/sj.neo.7900263 12407439 PMC1550339

[4] Cohen A Lim CS Davies AH. Venous thromboembolism in gynecological malignancy. Int J Gynecol Cancer. (2017) 27(9):1970–8. 10.1097/IGC.0000000000001111 28930804

[5] Nuyttens JJ Robertson JM Yan D Martinez A. The position and volume of the small bowel during adjuvant radiation therapy for rectal cancer. Int J Radiat Oncol Biol Phys. (2001) 51(5):1271–80. 10.1016/s0360-3016(01)01804-1 11728687

[6] Morgan MA Iyengar TD Napiorkowski BE Rubin SC Mikuta JJ. The clinical course of deep vein thrombosis in patients with gynecologic cancer. Gynecol Oncol. (2002) 84(1):67–71. 10.1006/gyno.2001.6452 11748979

[7] Harbin MM Lutsey PL. May-Thurner syndrome: history of understanding and need for defining population prevalence. J Thromb Haemost. (2020) 18(3):534–42. 10.1111/jth.14707 31821707

[8] Neglén P Berry MA Raju S. Endovascular surgery in the treatment of chronic primary and post-thrombotic iliac vein obstruction. Eur J Vasc Endovasc Surg. (2000) 20(6):560–71. 10.1053/ejvs.2000.1251 11136593

[9] De Maeseneer MG Kakkos SK Aherne T Baekgaard N Black S Blomgren L Editor’s choice-European society for vascular surgery (ESVS) 2022 clinical practice guidelines on the management of chronic venous disease of the lower limbs. Eur J Vasc Endovasc Surg. (2022) 63(2):184–267. 10.1016/j.ejvs.2021.12.024 35027279

[10] Ducasse E Calisti A Speziale F Rizzo L Misuraca M Fiorani P. Aortoiliac stent graft infection: current problems and management. Ann Vasc Surg. (2004) 18(5):521–6. 10.1007/s10016-004-0075-9 15534730

[11] Dosluoglu HH Curl GR Doerr RJ Painton F Shenoy S. Stent-related iliac artery and iliac vein infections: two unreported presentations and review of the literature. J Endovasc Ther. (2001) 8(2):202–9. 10.1177/152660280100800217 11357983

[12] Chambers ST. Diagnosis and management of staphylococcal infections of vascular grafts and stents. Intern Med J. (2005) 35(Suppl 2):S72–8. 10.1111/j.1444-0903.2005.00981.x 16271063

